# Quantifying neuro-motor correlations during awake deep brain stimulation surgery using markerless tracking

**DOI:** 10.1038/s41598-022-21860-7

**Published:** 2022-10-27

**Authors:** Anand Tekriwal, Sunderland Baker, Elijah Christensen, Humphrey Petersen-Jones, Rex N. Tien, Steven G. Ojemann, Drew S. Kern, Daniel R. Kramer, Gidon Felsen, John A. Thompson

**Affiliations:** 1 Department of Neurosurgery, University of Colorado School of Medicine, 12800 E. 19th Ave., Mail Stop 8307, Aurora, CO 80045 USA; 2 Department of Physiology and Biophysics, University of Colorado School of Medicine, 12800 E. 19th Ave., Mail Stop 8307, Aurora, CO 80045 USA; 3 Neuroscience Graduate Program, University of Colorado School of Medicine, Aurora, CO 80045 USA; 4 Medical Scientist Training Program, University of Colorado School of Medicine, Aurora, CO 80045 USA; 5 Department of Neurology, University of Colorado School of Medicine, Aurora, CO 80045 USA

**Keywords:** Computational neuroscience, Neuroscience, Movement disorders, Parkinson's disease

## Abstract

The expanding application of deep brain stimulation (DBS) therapy both drives and is informed by our growing understanding of disease pathophysiology and innovations in neurosurgical care. Neurophysiological targeting, a mainstay for identifying optimal, motor responsive targets, has remained largely unchanged for decades. Utilizing deep learning-based computer vision and related computational methods, we developed an effective and simple intraoperative approach to objectively correlate neural signals with movements, automating and standardizing the otherwise manual and subjective process of identifying ideal DBS electrode placements. Kinematics are extracted from video recordings of intraoperative motor testing using a trained deep neural network and compared to multi-unit activity recorded from the subthalamic nucleus. Neuro-motor correlations were quantified using dynamic time warping with the strength of a given comparison measured by comparing against a null distribution composed of related neuro-motor correlations. This objective measure was then compared to clinical determinations as recorded in surgical case notes. In seven DBS cases for treatment of Parkinson’s disease, 100 distinct motor testing epochs were extracted for which clear clinical determinations were made. Neuro-motor correlations derived by our automated system compared favorably with expert clinical decision making in post-hoc comparisons, although follow-up studies are necessary to determine if improved correlation detection leads to improved outcomes. By improving the classification of neuro-motor relationships, the automated system we have developed will enable clinicians to maximize the therapeutic impact of DBS while also providing avenues for improving continued care of treated patients.

## Introduction

**Figure 1:**
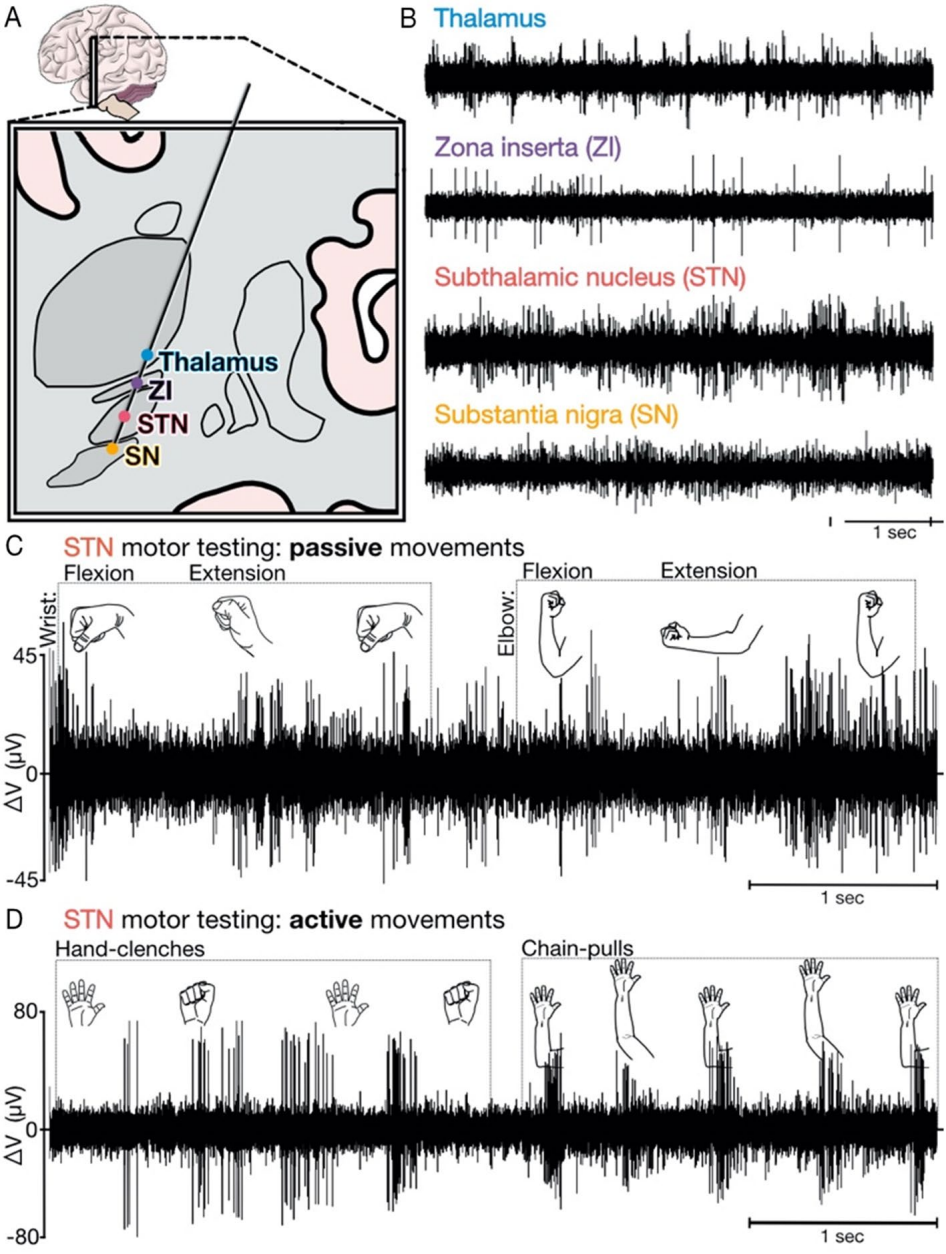
Microelectrode motor mapping. **(A)** Atlas schematic of a standard trajectory targeting the STN. (**B)** Regions traversed by electrophysiological recordings demonstrate unique activity profiles used to determine the optimal location to implant the DBS electrode. (**C&D)** Kinesthetic testing, both active and passive movements, aids in localizing motor regions targeted for DBS. Typically, MUA is audio-converted and clinicians listen for changes in activity while observing movements by eye. (**C)** Passive movements represent clinician-driven joint manipulation. Neural activity is modulated by flexion and extension of the wrist and elbow. (**D)** Active movements like hand clenches and chain pulls generally elicit larger changes in neural activity but are more difficult for clinicians to gauge as the timing of when movements occur is less directly under their control compared to passive movements. (Note: all components of figure are original work created by AT and designed within Affinity Designer, version 1.10.4).

Deep brain stimulation (DBS) is most commonly used for treatment of movement disorders, such as Parkinson’s disease (PD), with optimal implantation location often determined by the presence of kinesthetic-related neurophysiologic activity. Contemporary techniques, largely unchanged from those used in the 1960’s^1,2^, require clinicians to judge, in real-time, the presence or absence of changes in multi-unit neural activity (MUA) as patients are guided through movements (Fig. 1C,D). While useful for uncovering neuro-motor responses across targets and pathophysiologies, this process is subjective, labor intensive and can be challenging even for highly trained clinical teams. It also utilizes a small fraction of the information elicited by mapping neural structures^3^, which could be harnessed to identify neurophysiologic biomarkers useful in disease management (e.g., subthalamic (STN) beta-band power for PD^4^). Here, we demonstrate an intraoperative system which uses a deep learning assisted analytic pipeline to operationalize and improve upon standard motor mapping practices.

**Figure 2:**
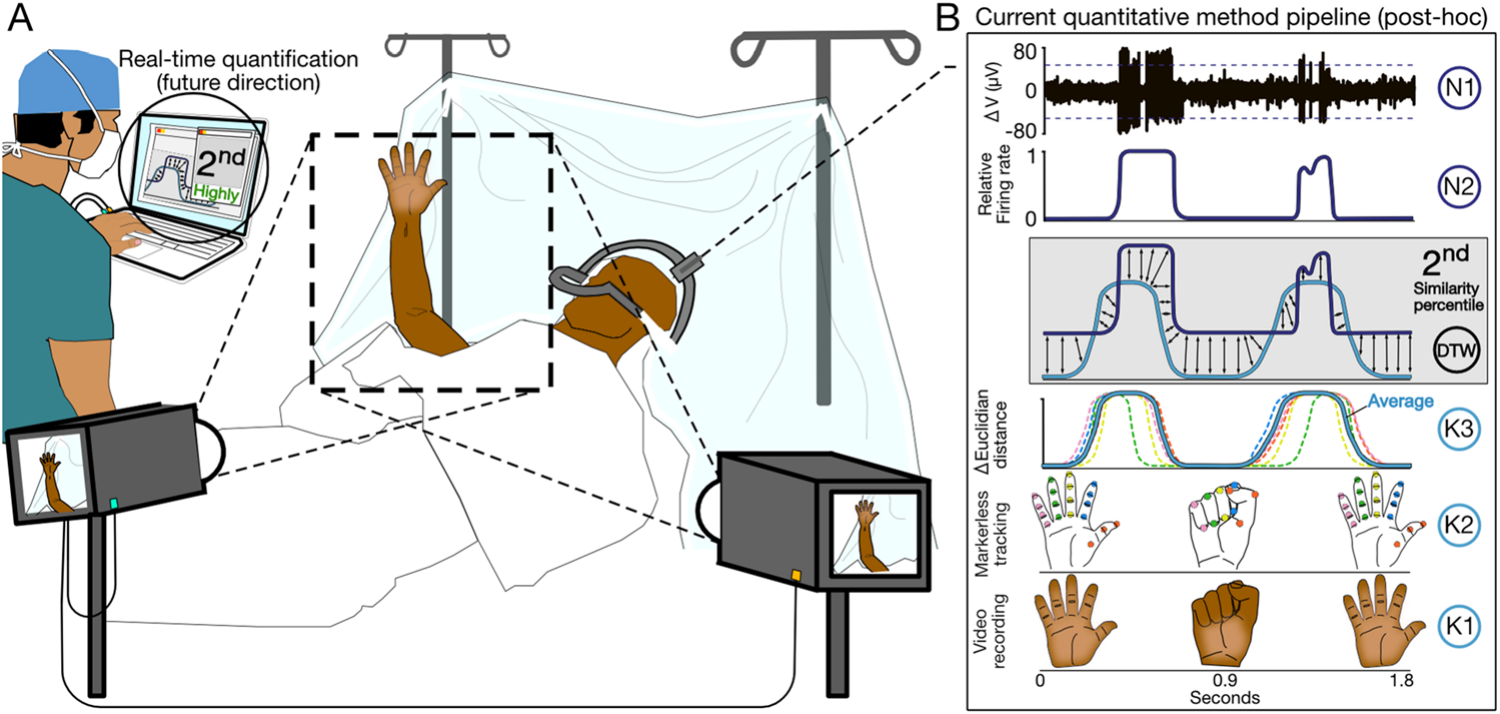
Intraoperative system and post-hoc data processing pipeline. (**A)** Schematic for intraoperative setup. Cameras with perpendicular views of motor testing area record videos that are synchronized with neural activity, recorded as part of standard clinical procedure, via a laptop, which can quantify neuro-motor correlations (see **B**) and report results to the clinician in real-time. While the analyses reported in this study are post-hoc (see **B**), computational costs are minimized to allow for real-time quantification of neuro-motor correlations for future deployment in clinical settings. (**B)** Overview of independent processing for neural (N, *top*) and kinematic (K, *bottom*) data along with example of using dynamic time warping (DTW, *middle*) to compare respective signals. Note that all data processing in current study occurs post-hoc. *N1*: Following acquisition by clinical electrophysiology recording system (see Supplementary Methods), voltage traces are further filtered to remove line noise and artifacts (see Materials and methods for details). Multi-unit activity (MUA) determined by thresholding (see Materials and methods for details). *N2:* MUA normalized to common scale (0 to 1) as kinematic data. *K1*: Motor testing epochs are identified and cut with frames extracted from the camera with optimal perspective. *K2*: Pre-trained neural network (see Materials and methods for details) extracts key regions of interest and applies digital tags to pre-identified anatomic features that are tracked for duration of recording. Here, anatomic features corresponding to a given finger share a common color. *K3*: Kinematic measures (here, change in Euclidian distance) are derived from each tracked feature, then subsequently averaged with related tracked features (i.e. common digit), smoothed, and normalized to common scale (0 to 1) as MUA. Dotted lines correspond to smoothed and averaged output for a given digit. Overall average shown as solid, teal line. *DTW:* Equal length and time-locked N and K signals are compared using DTW, which measures the Euclidian distance between each point on one signal with the most similar point in a given window on the corresponding signal (see Materials and methods for details). Lower measures of Euclidian distance correspond to comparisons of more similar signals. An overall measure of signal similarity (i.e. 2nd percentile) may be generated by comparing against a null distribution (not shown, see Fig. 3C) with similarity percentiles closer to 0 representing more similar signals. (Note: all components of figure are original work created by AT and designed within Affinity Designer, version 1.10.4).

**Figure 3:**
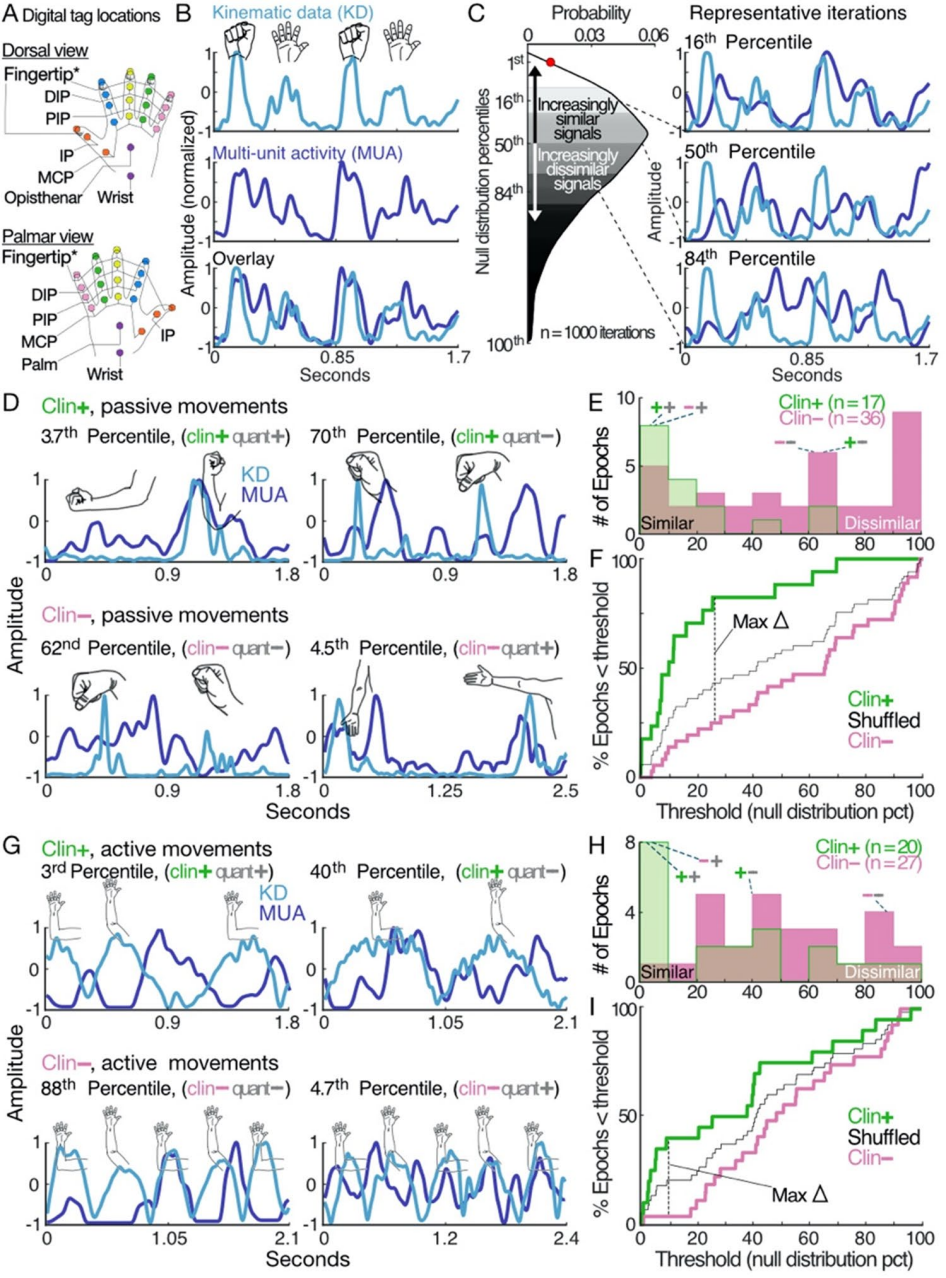
Determining significance of neuro-motor quantifications and comparing with clinical assessments. (**A)** All labeled and tracked points on the hand. For all presented analyses, frame-to-frame Euclidian displacement was averaged for the five fingertips as this was the minimum number of points needed for sufficient kinematic resolution. (**B)** Visualization of kinematic (top) and MUA (middle) signals for two cycles of hand clenches, following re-sampling to 1200 Hz and normalization. Overlay of the two signals (bottom) qualitatively demonstrates high similarity. Quantitative comparison of signal similarity using dynamic time-warping (DTW) finds a value of 89.1 a.u. for the sum of sample-to-sample comparisons between extracted kinematic and MUA signals. To determine statistical significance, the null distribution (n = 1000 iterations) percentile corresponding to 89.1 a.u. was calculated. Quantitative comparison to null distribution finds the relationship to be highly significant (1st percentile). (**C)** Null distribution used for comparison in B. The less the sample-to-sample distance between signals, the more similar signals being compared are. Hence, percentile values closer to 0 represent more similar signals whereas percentile values closer to 100 represent increasingly dissimilar signals. This is demonstrated with the representative iterations (i.e. lower percentile-value comparisons appear more similar on visual inspection). (**D)** Range of passive movement examples grouped by clinician (clin) assessment: *top row*, clin + (green); *bottom row*, clin- (pink). Percentile values in the subplot titles are the scalar output from the automated system analysis described in B & C. Percentile-values are compared against a 5th percentile threshold (i.e. “quant + ” if percentile-value < 5, “quant −” if percentile-value > 5). Quantitative determination is represented by the grey + or − symbols in the subplot titles while clinician assessment is represented by the green + or pink − symbols. (**E)** Percentile distributions shown separately for clin + (green) and clin − (pink) passive movement epochs. Values closer to 0 indicate comparisons wherein kinematic and MUA signals were quantified as similar by the automated system whereas values closer to 100 indicate dissimilar comparisons. Representative examples in D are shown in relation to the overall distribution of percentile-values and are identified by the notation (clin +/−, quant +/−), as described in D. (**F)** A receiver operator curve-like analysis for distributions shown in E. The “shuffled” group represents the rank ordered average over 1000 iterations of randomly assigning labels of clin + or clin − to epochs and their corresponding percentile-values. As expected, the shuffled group falls between the clin + and clin − lines. The maximum difference between the latter falls at the 26th percentile. (**G)** As in D, but for active movements. (**H)** As in E, but for active movements. (**I)** As in F, but for active movements. Maximum difference between clin + and clin − falls at 9.5th percentile. When compared to the differences noted in F, both the separation between groups and proportion of clin + epochs ruled in are greater for passive movements, but above chance performance for both movement types. (Note: all components of figure are original work created by AT and designed within Affinity Designer, version 1.10.4).

The key feature of the intraoperative system we describe (Fig. 2) is the quantitative approach for how MUA is objectively and reproducibly compared with patient kinematics. In recognition of this importance, the work presented herein focuses on providing a detailed description of how MUA and patient kinematics are extracted from intraoperative microelectrode and video recordings, respectively (Fig. 2), and most importantly, the method used to quantify signal similarity (Figs. 2B, 3B–C). The post-hoc comparisons we present (Figs. 2, 3) are the first applications of computer vision to clinical motor mapping procedures and provide a firm basis for further studies, including measuring impact on clinical outcomes and exploration of real-time comparisons with expert clinician assessments.

## Materials and methods

We collected intraoperative neural and video recordings from patients undergoing unilateral STN DBS surgery for treatment of PD at the University of Colorado Anschutz Medical Campus (AMC). Our study was approved by Colorado Multiple Institution Review Board (COMIRB) (Study protocol #16–1060). Written informed consent was obtained from all 7 study subjects (6 male; age 59 ± 11, PD duration of 8 ± 2 [years ± SD]) prior to surgical date in accordance with the COMIRB and Declaration of Helsinki protocols. Refer to Supplemental Methods for a more detailed explanation of participant recruitment and demographic information. Electrophysiologic recordings were captured as part of standard motor mapping as previously described^5,6^. Briefly, a recording electrode was advanced from 25 mm above and subsequently through the STN (Fig. 1A) with clinicians corroborating anatomic location with region-specific MUA signatures (Fig. 1B)^7^. In accordance with our institution’s typical STN DBS procedure, motor testing occurred at intervals of 0.3–0.5 mm, both by patients passively allowing clinicians to flex and extend limb joints (passive, Fig. 1C) and by patients actively and independently repeating a movement (active, Fig. 1D). Given systematic differences between passive and active testing, subsequent analyses distinguish between each. The extent of motor testing was determined by factors including clinician confidence in the presence (clin +) or absence (clin −) of neuro-motor relationships. Videos of testing were acquired using two cameras (Blackfly USB 3.0) connected to a laptop which controlled image data collection via a FLIR-Spinnaker-Python software development kit and user-generated Python graphical user interface (AMC IDEA Core). Cameras were placed on monopods (Avella A324D Aluminum 67″) near the foot and side of the bed to ensure at least one clear view of the tracked hand. Videos were collected for 10–30 s at each stopping point throughout the mapping procedure (average 36 videos/subject). While all analyses described hereafter were performed post-hoc, we have minimized computational cost whenever possible to optimize for real-world intraoperative use.

Kinematic data were extracted utilizing standard procedures developed as open-source tools within the DeepLabCut suite. All neural network training and testing was conducted with Python based software packages (DeepLabCut, TensorFlow) run in Linux. Unique models were constructed via DeepLabCut v2.2b6 with an automatic, kmeans clustering extraction method to isolate a subset of captured frames. Such frames were manually labeled to identify 21 visual anatomical landmarks of the hand both ventral and dorsal, including the base of the palm, center of the palm, metacarpophalangeal joints, proximal interphalangeal joints, distal interphalangeal joints, and tips of all digits (Fig. 3A). Among the cases, the total number of frames ranged from 14,400 to 50,400 (μ = 29,900), and via kmeans clustering, between 700 and 912 frames were selected per case for manual labeling (μ = 764, 3.22% of total). Various parameters were noted and/or adjusted prior to network training. The *Training Fraction* parameter, which defined a fraction of the network data to training and testing was set at 0.95, indicative of 95% reserved for iterative training. The pretrained *ResNet-50* was employed for all trainings, which contains 50 iteratively trained layers in object identification and probability density mapping for accurate tracking, demonstrating efficacy given a very small RMSE (3.09 ± 0.04) and accuracy with datasets as small as 200 frames^8^.

The *batch_size* parameter was set at 1, which established the computational memory needed in each iteration of training, meaning only one frame was to be analyzed at a time. This parameter was compatible with the *default_augmenter* “imgaug,” chosen given its robust filtering and smoothing of missing or skewed labels when paired with a 2D median filter to yield superb tracking among all videos. Additional information on the recommended default module “imgaug” and alternative options is available in the DeepLabCut user guide located on Github (https://github.com/DeepLabCut/DeepLabCut/blob/master/docs/standardDeepLabCut_UserGuide.md). The *p-cutoff* variable was raised from 0.01 to 0.06 as recommended in Mathis et al.^8^, as it serves to establish the likelihood threshold of distinguishing background from points of interest since the labeled points of interest were small. Lastly, *pos_dist_thresh* was lowered from 17 to 13 per recommendations in Mathis et al.^8^ given small fields of interest akin to *p-cutoff*. These models were trained until a plateau was reached in the network performance as measured by Huber loss to yield the smallest root mean square error, in which training iterations ranged from 195,000 to 268,100 (μ = 224,250). Networks achieved proficient performance as defined by > 95% accuracy of labeled test frames. In instances where accuracy remained below the > 95% threshold despite best efforts, sessions were not considered for further evaluation. Under such standards, among all cases, video samples deemed viable increased from 28.57 to 100% given adjustment of aforementioned parameters and iteration count, representing a wealth of usable data (μ = 71.55%, 213,977 total frames). No further refinement of each individual dataset was needed given the robust nature of the training, parameters, and inputted data. Frames were discarded if clear line of site was obscured, either by a clinical team member passing between the camera and tracked limb or during passive motor testing epochs in which clinicians’ hands occasionally occluded the tracked anatomic features. For additional descriptions of neural network training please refer to Mathis et al., 2018, 2020^8,9^.

Following extraction of markerless tracking data from video files, data were exported to Matlab wherein kinematic measures including Euclidean distance, cosine similarity, velocity, and acceleration of each labeled point were calculated. For consistency and to simplify our analyses, we utilized Euclidean distance as our primary kinematic variable for all results. To compare kinematic data acquired at 60 Hz with MUA acquired at 44 kHz, each were resampled to 1200 Hz. This frequency was chosen as it added minimal noise when upsampling kinematic data and preserved the most relevant information contained within the electrophysiologic signal since we expected to record at most 3 neurons per lead with firing rates < 125 Hz, thereby staying comfortably within 1200 Hz. Matlab function *resample* was utilized as this afforded an anti-aliasing method to altering sampling frequency. With respect to kinematic data, artifacts from temporary loss of tracked points were detected using a 4.5*SD threshold. Within windows identified as artifactual, kinematic data was locally interpolated to maintain underlying structure of movements.

Following acquisition of electrophysiology data as part of routine clinical procedure, data was processed post-hoc offline. Matlab 2020b (Mathworks, Natick, MA, USA) was used for neural data series data analysis and comparisons of neural to kinematic data described in the manuscript. Selecting the most appropriate filters for analyzing multi-cell neural data is critical to efficient extraction of spikes^10^. For pre-processing specific to MUA, time series voltage data was zero-lag filtered (Matlab function *filtfilt*) with a 2nd order Butterworth bandpass filter between 100 and 4000 Hz normalized to the Nyquist frequency (22 kHz). To establish a threshold for spike detection which would not be inflated by anticipated high firing rate MUA, an estimate for the standard deviation of background noise (SD_bn_) was calculated by taking the median of the absolute value of the band passed signal divided by 0.6745^10,11^. Beginning with a 4.5* SD_bn_ threshold, average firing rate was estimated within a possible range of 2.5* SD_bn_ to 6.5* SD_bn_ until an average firing rate of > 80 Hz and < 400 Hz was achieved or the limits of the range met. Firing rate thresholds were determined based on expected STN firing rates (80–125 Hz) and expected number of neurons per recording (1–3 neurons). This dynamic but constrained threshold was employed to account for varying levels of signal to noise (spike to background) found between recordings taken at different depths and across subjects. Due to the multi-unit nature of recordings, no inter-spike interval was imposed upon threshold crossings. To maintain the pattern of detected spikes when down sampling from 44 kHz to 1200 Hz for comparison with kinematic data, timestamps were divided by 44/1.2 and rounded to the nearest integer values. In cases where this process resulted in multiple instances of a down sampled timestamp value, only one representation was retained. Next, instantaneous firing rate was calculating for each sample point (1200 Hz) as the reciprocal of the interval between two spikes surrounding a sampled time point.

Comparisons between kinematic and MUA signals were conducted using dynamic time warping (DTW; Matlab function, *dtw*). This method optimally matches two temporal sequences by mapping each individual point on one signal with a point on the corresponding signal that minimizes the Euclidian distance separating the signals. Comparisons characterized by smaller Euclidian distance measures are more similar than comparisons with large measures, assuming equal length signals. A key constraint of DTW requires each point on a signal to match with at least one point on the other with mapping of indices monotonically increasing. As kinematic and MUA signals compared with DTW were resampled to a common frequency and length, overall signal lengths were not warped to align with one another, but in point-to-point comparisons the temporal relationships could dynamically adjust within a set window (200 ms). Rationale for use of DTW, as well as how statistical significance was determined for resulting comparisons, is elaborated upon in the Results section.

## Statistics

All reported statistics are 2-tailed with the appropriate parametric or nonparametric test applied following normality testing using the Lilliefor’s test (Matlab function, *lilietest*). The analysis pipeline we describe was iterated upon and for each specific analysis such as the use of dynamic time warping, a closely related technique like cross-correlation was used to confirm findings. In cases where experimenters selected for variables like window size when smoothing signals or related methods, parameters were varied and again confirmed to have minimal effect on outcomes.

Comparisons between quantitative and clinical assessments (Fig. 3D–I) were carried out once the quantitative method was standardized to a uniform pipeline optimized for all available datasets (n = 7 patients). Parameters were not subsequently optimized to yield findings more supportive of the quantitative method in head-to-head comparisons, i.e. all reported findings reflect initial comparisons.

## Results

First, movement kinematics were extracted from videos using a deep neural-network trained to identify and track anatomic features (Fig. 3A)^8^. In total, we tracked 21 points on the hand (Fig. 3A) with an average mean reprojection pixel error of 0.0244, indicative of exceptionally high accuracy^8^. Kinematic information was derived by measuring the frame-to-frame Euclidian distance for each tracked point. After our initial assessment across all cases, we determined that sufficient positional information could be derived from averaging across the 5 fingertips and thereafter limited our kinematic extraction to these points. MUA was isolated from neural activity using a 4.5 standard deviation threshold, and after processing to a common sampling frequency (1200 Hz) and normalizing amplitudes, compared to kinematic data. For additional details on data collection and processing, refer to Supplementary Methods. Signal similarity was determined by summing the distance between successive sample points of one signal with the most similar point in the other within a set window, a technique known as dynamic time warping (DTW; very similar results were also obtained using cross-correlation [Matlab, *crosscorr*]). Of the comparative methods we tested, DTW was best able to accommodate the variable amount of latency between neuro-motor signals for relatively low computational cost. In our analyses, we employed a relatively liberal 200 ms window to account for extended physiologic signal latency secondary to PD pathophysiology^12–15^. To determine the statistical strength of a given DTW measurement (Fig. 3B, bottom), we referenced against a boot-strapped null distribution, which was generated by iterative comparisons (n = 1000) between the retained kinematic signal (Fig. 3B, top) and unique MUA segments. MUA segments were randomly selected from a database composed of STN MUA recordings aggregated from all study participants (Fig. 3C). We then calculated a similarity percentile value by determining where along the null distribution the initial comparison of interest fell, with percentiles closer to 0 indicating more similar signals (Fig. 3C). Because the kinematic signal is retained between a given comparison and iterations comprising the corresponding null distribution, percentile endpoints are particularly robust measures. The similarity between the example signals in Fig. 3B lies at the 1st percentile (Fig. 3C, red circle), indicating exceptionally high similarity unlikely to occur by chance.

To compare the automated system’s assessments with clinicians’, we identified which neuro-motor relationships were clearly described in the surgical case notes as similar (clin +) or dissimilar (clin −) (100/117 epochs). Expert clinician assessments relied on audio and visual inspection of neural activity correlated with active and passive range of motion, the same signals used by the quantitative approach. Clinicians incorporated additional information to determine final implant location, including imaging and local field potential (LFP) data, that did not contribute to neuro-motor determinations. For epochs with high quality clinician notes (n = 100), we next quantified signal similarity; percentile values < 5 were categorized as having significantly similar kinematic and MUA signals (quant +) and percentile values > 5 as dissimilar (quant −). Our dataset of 100 epochs contained all combinations of classifications (clin + quant + , clin − quant −, clin + quant −, clin − quant +, Fig. 3D,G). When assessments agreed, we were highly confident in the validity of the shared determination (+ + , Fig. 3D,G top left; – , Fig. 3D,G bottom left). For examples found to be clin + but quant − (+ −, Fig. 3D,G top right), kinematic and MUA signals demonstrated consistent phasic relationships, but at non-physiologic latencies (i.e. > 200 ms). The automated system removes the possibility such signals could be interpreted as similar by limiting the window for DTW and comparing against the null distribution, tasks that are exceedingly challenging to accomplish without computational tools. The reciprocal observation can be made for epochs gauged as clin − but quant + (− + , Fig. 3D,G bottom right); signal profiles appear uncorrelated but have similar structure when accounting for dynamic shifting. Taken together, representative comparisons of quantitative and clinician assessments are often aligned, and when not, it seems likely clinical determinations would bend towards those arrived at by the automated system if clinicians could integrate data on features like signal latency into their decision-making.

To test if these anecdotal observations were reflected in the group data, we analyzed the percentile-value distributions for clin + compared to clin − epochs. To compare the structure of the distributions, we utilized a shift function, a paired analysis between the matched deciles (10th, 20th, 30th… 90th percentiles) of two distributions^16^. For passive movements, the clin + and clin − distributions significantly differed with respect to central tendencies (*p* = 9.7 × 10–5, Wilcoxon rank-sum test) (Fig. 3E), and more specifically, between deciles corresponding to the 30–90th percentiles (7/9 deciles). This indicated the automated system generally found that clin − epochs contained dissimilar signals (i.e. percentile-values close to 100, Fig. 3C) and clin + epochs contained similar signals (i.e. percentile-values close to 0, Fig. 3C), aside from a minority of clin − epochs which the automated system judged to have highly similar kinematic and MUA signals. Comparisons for active movements were also significantly different (*p* = 0.032, t-test) (Fig. 3H), but with an inverted pattern of significant differences in deciles (10–30th, 3/9 deciles). This also suggested general agreement between clinician and automated system assessments for active movements. However, many clin + epochs were determined by the automated system to contain dissimilar signals.

While we expected the automated system to proficiently differentiate between clin − and clin + epochs for both active and passive movements, it was notable that the shift function analyses found nearly no overlap in which deciles differed. To determine if this may be due to a systemic difference in how movement types were quantified, we compared the distribution of percentile-values for all passive (n = 58) and active (n = 59) epochs and found no significant difference between any deciles, nor when comparing the central tendencies of each distribution (*p* = 0.66, Wilcoxon rank sum test). Another plausible explanation relates to the differences in how passive versus active motor testing occurs. For passive movements, clinicians have a high level of control on precisely when movements occur because they are physically moving the patient’s limb themselves. As a result, clinicians can adeptly rule out when movements do not relate to changes in MUA by stochastically initiating movements to see if the pattern of MUA is altered in kind. However, given that neuronal recruitment is generally less for passive than active movements (Fig. 1C amplitude < Fig. 1D) and clinicians are limited in the intensity they can comfortably drive patients to passively execute, there may be epochs classified as clin − wherein a meaningful but subtle relationship is present. This would account for the minority of clin − epochs found to contain highly similar signals by the automated system. Conversely, active movements likely elicit greater neuronal activity in a non-specific manner, the endogenous rhythmicity of which may result in classification of epochs as clin + when the observed relationship is happenstance. If true, this explanation accounts for the many clin + epochs found by the automated system to contain dissimilar signals.

To investigate these inferences, we quantified the percentile-value threshold which best distinguishes between clin + from clin − groups with respect to the automated system’s findings (Fig. 3F,I). For passive movements (Fig. 3F), a threshold of percentile-value = 26 ruled in 82% of clin + and 25% of clin − epochs (*p* = 0.00011, odds ratio = 14, Fischer exact test), which supports our inference that passive movements are a sensitive test of neuro-motor relationships. For active movements (Fig. 3I), the identified threshold was percentile-value = 9.5, with 40% of clin + and 4% of clin − epochs ruled in (*p* = 0.0026, odds ratio = 17, Fischer exact test). This high specificity aligned with the insight that active movements provoke strong neural responses, but at this relatively conservative threshold less than half clin + epochs are identified as quant + and correspondingly there were few clin − epochs determined to be quant + . However, at the second most ideal threshold of percentile-value = 41, 70% of clin + and 34% of clin − epochs were ruled in (*p* = 0.0129, odds ratio = 5, Fischer exact test) which illustrates a proportionally high number of clin − epochs determined as quant + . Taken together, these findings support our insights on the relative strengths and weaknesses of passive and active testing.

## Discussion

The novel computer vision assisted neuro-motor assessment system presented here represents a significant step forward in combining clinical best practices with advanced machine learning methods. We have shown that intraoperative kinematics extrapolated from video-captured motor testing and subsequently compared with neural activity can produce objective measures of neuro-motor signal similarity. Further development of this approach would facilitate integration into current clinical workflows to potentially improve treatment efficacy. As a preliminary evaluation of the clinical utility of this system (Fig. 2), we compared our quantitative approach against expert clinical assessment as the latter is our best “ground truth” measure for gauging neuro-motor signal similarity extrapolated from intraoperative data.

Although our quantitative approach performed favorably in this direct comparison, further research to determine impact on treatment efficacy could focus on assessing the relationship between regions of STN that evince increased strength of neuro-motor relationships with established biomarkers for optimal STN implant target, like maximum beta frequency peak^17–19^. Furthermore, in the post-operative programming setting, follow-up studies contrasting symptom severity when stimulating at sites where similar or dissimilar neuro-motor signals were recorded would directly link a quantitative intraoperative insight with clinical impact. Similarly, comparisons when stimulating at locations where expert versus quantitative methods indicated high levels of neuro-motor signal similarity are well suited to this setting. In addition to informing the utility our quantitative approach may have, such studies would also provide insight into the utility of motor mapping as part of awake procedures itself.

In parallel, findings from programming studies can be used to retrospectively inform optimal percentile thresholds for determining whether correlations are clinically informative or not. Such information can only be determined by assessing clinical endpoints, which will be highly instructive to optimizing future iterations of automated systems. Although the automated system we describe determines the strength of neuro-motor relationships post-hoc, increased processing power for the intraoperative computer would allow it to run in near real-time, which would be necessary to inform site targeting during DBS surgery. The most significant technical barrier to deploying such a system is adequate training of a more generalized upper limb tracking model, but preliminary efforts indicate that this is surmountable with a large enough training dataset. By staging studies in this way, the balance between exploring advances in clinical care and supporting the efficacy of existing targeting approaches may be maintained.

Adopting a quantitative approach to assessing neuro-motor relationships may further operationalize and enhance clinical classification of motor symptoms by increasing the precision of well-validated methods such as the MDS-UPDRS (Movement Disorders Society Unified Parkinson’s Disease Rating Scale)^20,21^. Application of markerless tracking techniques to the quantification of motor symptom severity has the potential to evolve motor assessments from clinician administered rating scales to precise and automated measurement of kinematics captured in real-world scenarios^22,23^. Doing so as part of post operative programming sessions is one natural extension of the quantitative approach we have described. Current best practices require patients to spend hours in the clinic, repeating often tedious movements under different stimulation settings while specialists score movements to identify optimal parameters. In addition to being time and resource intensive, this approach underutilizes the capabilities of modern devices. Stimulatory probes with additional contacts and current steering capabilities offer hundreds of different stimulation montages, but only a handful of options can be tried at in-clinic programming session. Research efforts using external sensors like accelerometers and related assessment tools have shown current symptom assessment and programming practices can be improved upon^24–27^. However, sensor-based methods do not capture as broad a range of symptoms as markerless tracking methods may, nor can they be incorporated into clinical practice as seamlessly. For example, markerless tracking may be used to capture patients’ movements at home using their own smart phones, allowing care to be tightly titrated and personalized to patients’ evolving needs without the need for additional hardware. In carrying out such work, clinicians and researchers could begin to construct an evidence-based treatment plan that incorporates neuro-motor relationships into diagnosis and disease management. It is highly likely that biomarkers of disease subtypes, like tremor- or akinetic-dominant PD, can be identified by leveraging deep learning tools. Our study’s dataset included two patients characterized as mixed sub-type tremor dominant, their subtype diagnosis evident in both kinematic and neural recordings. Although we lacked the statistical power to investigate whether distinct neuro-motor relationships described tremor dominant subtypes, reports by other groups suggest subtype specific targets likely exist^28,29^ which may be best identified through categorization of the neuro-motor relationships described in this report. A similar comparison between the six early-onset PD cases with the singular typical onset case did not yield apparent qualitative differences, likely due in part to limited sample size and PD sub-type heterogeneity. As advances in closed loop stimulation based on gating stimulation to beta-band strength have shown, such discoveries can catalyze meaningful improvements in treatment efficacy.

In summary, the quantitative approach for intraoperative motor mapping we have developed is well adapted to the challenges functional neurosurgical teams face and holds significant promise for not only improving outcomes on a surgical case by case basis, but in understanding pathophysiology of diseases like PD. This is in addition to being accessibly packaged and designed to cleanly integrate with current practices, affording clinicians the opportunity to provide a higher quality of care to patients otherwise refractory to care. As we have described, further studies are needed to confirm our approach can contribute to improved clinical outcomes, but our system is well suited for these next steps. Beyond the immediate implications of our work on awake motor mapping procedures, we have demonstrated that highly specialized clinical skills may be adjunctively supported by careful application of machine learning techniques. In addition to potentially improving clinical outcomes, approaches like our quantitative method have the potential to democratize access to high levels of care currently available only in specialized settings like university hospitals.

## Supplementary Information

Supplementary Information.

## Data Availability

The datasets used and/or analyzed during the current study available from the corresponding author on reasonable request.

## Abbreviations

DBS: Deep brain stimulation
PD: Parkinson’s disease
MUA: Multi-unit activity
STN: Subthalamic nucleus
SD: Standard deviation
COMIRB: Colorado Multiple Institution Review Board
DTW: Dynamic time warping
AMC: Anschutz Medical Campus
IDEA: Innovation and Design for Experimentation and Analysis
