# Supplementary material for: Quantifying neuro-motor correlations during awake deep brain stimulation surgery using markerless tracking
Source: Sci Rep. 2022 Oct 27;12:18120. doi: 10.1038/s41598-022-21860-7 (PMC9613670; doi:10.1038/s41598-022-21860-7)
Supplement: Supplementary file 1 — Supplementary Information. [file 41598_2022_21860_MOESM1_ESM.docx]

**Supplemental Methods**

**Data collection:**

Neural recordings were acquired from DBS surgeries using the NeuroOmega system (Alpha Omega Engineering, Nazareth Israel). Raw and spiking signals were sampled at 44 kHz by a 16-bit A/D converter (using ±1.25V input range, i.e., ∼2 µV amplitude resolution) and band-passed from 0.7 to 9000 Hz using a hardware four-pole Butterworth filter. MER began at 25 millimeters above the ventral border of STN and advanced in steps of 100-1000 micrometers.

As described in the manuscript, videos of testing were acquired using two cameras (Blackfly USB 3.0) connected to a laptop which controlled image data collection via a FLIR-Spinnaker-Python software development kit and user-generated Python graphical user interface (AMC IDEA Core). Cameras were placed on monopods (Avella A324D Aluminum 67”) near the foot and side of the bed to ensure at least one clear view of the tracked hand. Videos were collected for 10-30 seconds at each stopping point throughout the mapping procedure (average 36 videos/subject).

**Data exclusion:**

Two cases originally included in our study were excluded from analysis because those DBS surgeries targeting the GPi for implantation as opposed to the STN. Initially, we included these cases in our analyses, which were not meaningfully affected, but opted to remove these cases to provide a more unified study dataset.

**Recruitment and demographic information**

Patients were recruited at the University of Colorado Anschutz Medical Campus through the Movement Disorders Center from the population of adult patients undergoing deep brain stimulation (DBS) surgery for treatment of PD. Surgical candidacy was assessed by a clinical panel composed of representatives from neuropsychology, neurology, neuroradiology and neurosurgery. During the time period when the study was actively collecting data, all potential subjects were invited to participate in the study. Given that our study was observational with all processing and analysis performed after the clinical event, there were no significant exclusion criteria for patients as the potential study participants were already deemed appropriate candidates for DBS itself. All patients in our study had a primary diagnosis of PD without significant comorbidities and were treated with DBS targeting the subthalamic nucleus (STN). Patients were deemed appropriate candidates for DBS by clinical consensus, which included the determination patients were likely to receive several years of significant quality of life improvement following successful DBS treatment.

Participant 1: M, 68 at time of PD diagnosis with 7-year history at time of surgery

Participant 2: M, 57 at time of PD diagnosis with 9-year history at time of surgery

Participant 3: M, 40 at time of PD diagnosis with 5-year history at time of surgery

Participant 4: F, 52 at time of PD diagnosis with 9-year history at time of surgery

Participant 5: M, 41 at time of PD diagnosis with 11-year history at time of surgery

Participant 6: M, 56 at time of PD diagnosis with 8-year history at time of surgery

Participant 7: M, 39 at time of PD diagnosis with 10-year history at time of surgery

**Blinding**

Experimenters were blinded to the associated neural data when pre-processing and selecting for epochs of kinematic activity and similarly blinded to the associated kinematic data when pre-processing neural data. Reported statistics stemming from comparisons with boot-strap generated distributions were run once and that measure recorded without prior knowledge of the associated clinical determination.

**Code and Data availability**

Analysis code and corresponding data will be made available once the outcome of authors’ patent application, which encompasses the work described herein, is known. Until that time, code will be made available upon reasonable request.
